# Tensions and opportunities in social prescribing. Developing a framework to facilitate its implementation and evaluation in primary care: a realist review

**DOI:** 10.3399/BJGPO.2021.0017

**Published:** 2021-05-26

**Authors:** Sara Calderón-Larrañaga, Yasmin Milner, Megan Clinch, Trisha Greenhalgh, Sarah Finer

**Affiliations:** 1 Centre for Primary Care and Mental Health, Institute of Population Health Sciences, Queen Mary University of London, London, UK; 2 Bromley By Bow Health Partnership, London, UK; 3 Barts Health NHS Trust, London, UK; 4 Nuffield Department of Primary Care Health Sciences, University of Oxford, Oxford, UK

**Keywords:** social prescribing, primary health care, general practice, realist review, health policy

## Abstract

**Background:**

Social prescribing (SP) involves linking patients in primary care with services provided by the voluntary and community sector (VCS). Despite growing interest within NHS primary care, it remains unclear how and under what circumstances SP might contribute to good practice.

**Aim:**

To define ‘good’ practice in SP by identifying context-specific enablers and tensions. To contribute to the development of an evidence-based framework for theorising and evaluating SP within primary care.

**Design & setting:**

Realist review of secondary data from primary care-based SP schemes.

**Method:**

Academic articles and grey literature were searched for qualitative and quantitative evidence following the Realist And Meta-narrative Evidence Syntheses — Evolving Standards (RAMESES). Common SP practices were characterised in three settings (general practice, link workers, and community sector) using archetypes that ranged from best to worst practice.

**Results:**

A total of 140 studies were included for analysis. Resources were identified influencing the type and potential impact of SP practices and four dimensions were outlined in which opportunities for good practice arise: 1) individual characteristics (stakeholder’s buy-in, vocation, and knowledge); 2) interpersonal relations (trustful, bidirectional, informed, supportive, and transparent and convenient interactions within and across sectors); 3) organisational contingencies (the availability of a predisposed practice culture, leadership, training opportunities, supervision, information governance, resource adequacy, accessibility, and continuity of care within organisations); and 4) policy structures (bottom-up and coherent policymaking, stable funding, and suitable monitoring strategies). Findings were synthesised in a multilevel, dynamic, and usable SP framework.

**Conclusion:**

The realist review and resulting framework revealed that SP is not inherently advantageous. Specific individual, interpersonal, organisational, and policy resources are needed to ensure SP best practice in primary care.

## How this fits in

Despite widespread policy support and proliferation, evidence for the effectiveness of SP is methodologically weak. The realist review contributes to this much-needed evidence base by identifying how and under what circumstances this intervention might best be considered. These findings suggest that specific resources at individual, interpersonal, organisational, and policy levels appear to condition the type and potential impact of SP practices in primary care. The resulting framework may prove particularly useful for end users when implementing, adapting, and evaluating new or existing SP initiatives.

## Introduction

SP involves linking patients in primary care with services provided by the VCS.^1^ Community-based resources may be aimed at addressing employment, food insecurity, housing, or financial problems, as well as ‘healthy lifestyle’ interventions, such as cooking classes, weight management, or exercise programmes.^1^ The link between health and third sectors is often provided by a ‘social prescriber’ (also called ‘link worker’), whose role ranges from signposting to more intensive approaches involving patients’ needs assessments, ongoing support, and recommendations of relevant VCS services.^2,3^ Community-based activities are typically non-medicalised, provided locally, and more likely to be culturally appropriate to the local communities.^4^

SP is being widely adopted in the UK, including at policy level within the NHS Long Term Plan.^5^ Proponents of SP suggest it may contribute to address the social and behavioural determinants behind escalating burden of long-term conditions and health inequalities.^6^ It is also argued that SP could improve the efficient use of health and social care resources, by enhancing self-care and community support networks.^7^ However, recent systematic reviews have failed to prove consistent health, service utilisation, or cost benefits,^8–13^ in part owing to the use of methods and research designs not suited for the evaluation of such complex interventions.^14,15^ Further research is also needed on the applicability of SP to specific areas of health need and contexts.^16,17^

In this article, a realist approach is used to synthesise current evidence on SP implementation and delivery. Previous realist reviews on SP have mainly focused on the role of link workers in developing connections between stakeholders^18^ and identified some preconditions for enhancing patients’ enrolment, engagement, and adherence.^19^ However, no previous studies have investigated SP in the context of complex (and often conflicting) interpersonal (micro), organisational (meso), and social and policy (macro) relationships, nor their influence on service delivery. The review seeks to define ‘good’ practice in SP by identifying both context-specific enablers and tensions that may hinder efforts. Building on these findings the authors aim to synthesise a comprehensive, complexity-informed framework, which could potentially be used for theorising and evaluating SP in primary care and applied to areas of specific health need. The review is part of a broader empirical study and will be followed by a realist evaluation that will explore the applicability of the framework to type 2 diabetes prevention in populations at high risk.^20^

## Method

The review followed the RAMESES guidance.^21,22^ Realist reviews have the potential to extend beyond decontextualised analysis to explore and explain ‘why’, ‘for whom’, and ‘in what circumstances’ interventions might (or might not) work while following a systematic process.^22^ Explanations focus on mechanisms and the contexts required to trigger them, resulting in the development, refinement, and testing of context-mechanism-outcome configurations (CMOCs) (see Box 1 for definitions). This theory-driven, evidence-based methodology is increasingly being used in health services research and the evaluation of complex interventions such as SP.^23–25^

Box 1 **Definition of realist concepts**

Context:

Refers to the background of a programme. Pawson suggests that contexts can be understood and illustrated diagrammatically as a set of concentric ovals surrounding the intervention.^25^ He distinguishes the following four contextual layers: 1) individual characteristics and capacities of the various stakeholders involved; 2) interpersonal relationships between the stakeholders; 3) the institutional (or organisational) rules, norms, and routines local to the intervention; and 4) the wider social, cultural, and policy infrastructure. Some aspects of these contexts might enable (or hamper) particular mechanisms to be triggered.

Mechanism:

Refers to the resources and conditions, which operate in particular contexts to generate outcomes of interest. They are the ‘agents of change’, usually hidden and sensitive to variations in the context.

Outcome:

Refers to the intended, unintended, or unexpected impact or behaviours resulting from the interaction between mechanisms and contexts.

Context-mechanism-outcome configuration (CMOC):

It is a hypothetical explanation that the intervention works (or does not work) (O) because of the action of some underlying mechanisms (M), which only come into operation in particular contexts (C).

As summarised in Box 2, the review followed four iterative stages: 1) data searches; 2) study selection and quality assessment; 3) data extraction and analysis; and 4) data synthesis and conclusions. A detailed description of the review methods has been included in Supplementary Appendix S1 and published elsewhere.^20^

Box 2 **Realist review components**

Data searches:

Two distinct literature searches were carried out under the guidance of a specialist librarian. The strategy and databases for the main search are specified in Supplementary Appendix S2. In addition to database searching, citations contained in the reference lists of the articles included in the review were manually retrieved and grey literature resources were searched. The main search was reproduced by a second reviewer for consistency and discrepancies were solved by discussion. Based on the retrieved literature, policy-level mechanisms were identified (including drivers and contractual agreements) as in need of further exploration and refinement. An additional search was undertaken that focused on these specific areas by manually retrieving articles from the reference lists of relevant studies.

Study selection and quality assessment:

The review included all studies published in English, French, or Spanish on interventions linking adults (aged ≥18 years) in primary care with voluntary and community sector (VCS) organisations, regardless of study design (quantitative, qualitative, and mixed methods) and including all social prescribing (SP)-related outcome measures. Studies focusing on specific (sub)populations with special needs were excluded (for example, learning disabilities, sensory impairment, and cognitive impairment). The relevance, rigour, and richness of all studies included were assessed. For the main search, study relevance was accorded on the involvement of link workers within the SP intervention. For the additional search, studies were classified as highly relevant if they explored the organisational and policy environment within which interventions were commissioned and delivered. The methodological strength of included studies were also assessed using study design-specific validated tools (rigour), and the extent to which that source could contribute to the developing context–mechanism–outcome configurations (CMOCs) (richness). See Supplementary Appendix S3 for more details on the quality appraisal criteria used.

Data extraction and analysis:

The main reviewer developed conceptual diagrams and preliminary codes during an initial familiarisation stage, which focused first on the richest sources. The manual coding framework was then transferred into NVivo (version 10) and further tested and refined by applying it to the rest of the articles (deductively) or modifying it as needed to incorporate new findings coming up in the data (inductively). The analysis involved switching reflexively from data to theory as required and continued under a realist and explanatory logic: study outcomes were first defined using SP archetypes, and then it was identified how they responded to conditions and resources (mechanisms) available in specific environments (contexts). This analysis was repeated throughout the review, enabling broad sets of CMOCs to be built.

Data synthesis and conclusions:

Explanations were inferred and written down of why certain SP practices occur (abductive reasoning), which involved comparing and contrasting data from different studies (juxtaposition of data sources). Where findings across studies differed, further data were sought to identify explanations for why these differences occurred (reconciliation of disconfirming data). When findings across sources were consistent enough to develop patterns, they were incorporated into CMOCs (consolidation of sources of evidence). The CMOCs were further refined by re-scrutinising those already-included studies classified as highly relevant, conceptually rich, and rigorous. CMOCs were then synthesised in an initial framework that was further developed through iterative discussions within the research team.

The review was performed between September 2019 and May 2020, and was registered with PROSPERO. Throughout, the study objectives and emerging findings were shared and discussed with the stakeholder group, comprising health professionals, relevant members of the VCS and charitable organisations, and wider stakeholders (for example, related to clinical commissioning groups and Public Health England). Input from the stakeholder advisory group proved particularly relevant at the later stages (data synthesis and conclusions) to contrast and further refine the literature-based, theory-informed propositions with their practical real-world experience.

## Results

**Figure 1.**
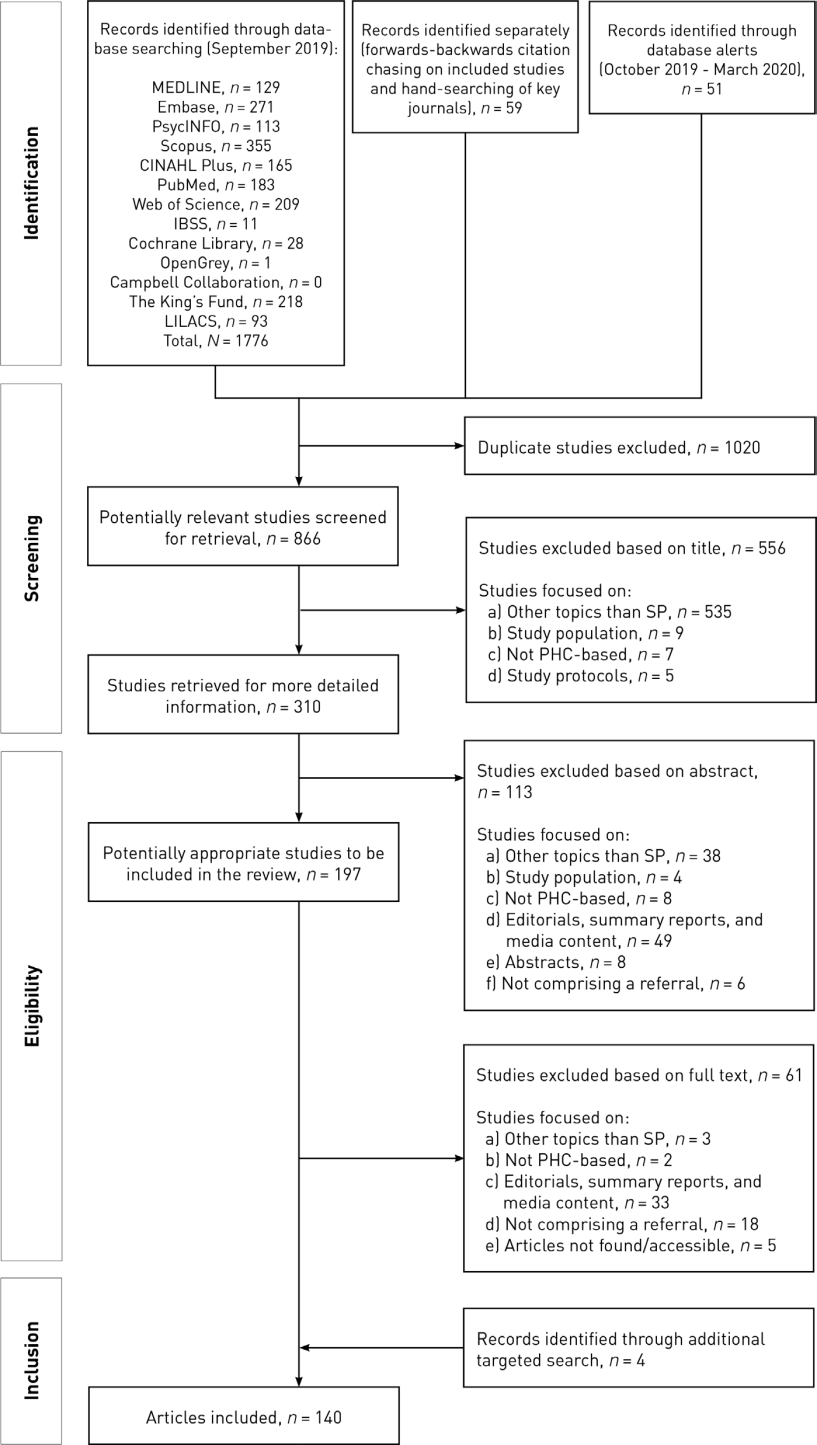
PRISMA flow diagram. PHC = primary health care. SP = social prescribing.

The PRISMA diagram (Figure 1) illustrates the screening and selection process in the evidence synthesis. Of the 140 references included in the review, 40 used qualitative methods, 37 used quantitative methods, 36 were mixed-methods studies, 25 were literature reviews, one SP research-based toolkit, and one evaluability assessment study. The quality appraisal resulted in 21 studies being classified as highly relevant, conceptually rich, and rigorous. The characteristics and quality of the studies included are further described in Supplementary Tables S1a–S1d and S2a–S2d, respectively.

### Study outcomes: SP archetypes

Following the analytic approach proposed by Papoutsi and colleagues,^26^ the first stage of the realist analysis involved defining the outcomes: ‘good’ practice in SP. This was undertaken through the identification of SP archetypes across three settings: general practice, link workers, and the VCS. Within each setting, common SP practices were identified, which were then grouped into six different evidence-based, theory-informed SP archetypes (two per study setting).^27,28^ Archetypes in each setting were conceptualised along a spectrum (ranging from best to worst practice) in order to encompass the range of phenomena observed in the reviewed literature and the exposure of tensions within service delivery and implementation.

#### 1. General practice: ‘holistic’ versus ‘fragmental’ SP archetypes

In a ‘holistic’ SP model, general practice workers understand, consider, and integrate patients’ social needs, and recommend community resources with the collaboration of other stakeholders (for example, link workers). Within a ‘fragmental’ model, patients’ needs (including ‘non-medical’ ones) are demarcated, triaged, and allocated to appointed stakeholders (for example, link workers). In this fragmental model, clinicians might become less aware of their patients’ wider social and community context, and hence unable to provide integrated, contextualised, high-quality clinical care.^29,30^ Therefore, ‘holistic’ SP was considered best practice.

#### 2. Link workers: ‘relational’ versus ‘transactional’ SP archetypes

Within a ‘relational’ model, ongoing and open-ended interactions allow link workers to assess, adapt, and respond iteratively to patients’ ever-changing needs, and make appropriate recommendations. In contrast, within a ‘transactional’ model, the service to be exchanged (for example, assessment of patients’ needs and referral to community-based interventions) has pre-established limits (for example, a maximum of six sessions with a ‘wellbeing coordinator’^7^), resulting in a lack of dynamism and flexibility that may hinder the co-production and customisation of care provision.^31,32^ Therefore, ‘relational’ SP was considered best practice.

#### 3. The voluntary and community sector: ‘redistributive’ versus ‘non-redistributive’ SP archetypes

A ‘redistributive’ SP model involves a varied and sustainable local community network able to address the diverse and changing priorities and issues (including social and economic) of those patients in greatest need. Within a ‘non-redistributive’ model, the availability of SP services varies inversely with the need of the population served. Murphy *et al*, for instance, showed how uptake and adherence to an exercise on prescription scheme was systematically lower among non-car owners in deprived areas.^33^ SP interventions risk exacerbating health inequalities when access is contingent on certain social or economic conditions.^34,35^ Therefore, ‘redistributive’ SP was considered best practice.

### Contexts and mechanisms

**Figure 2.**
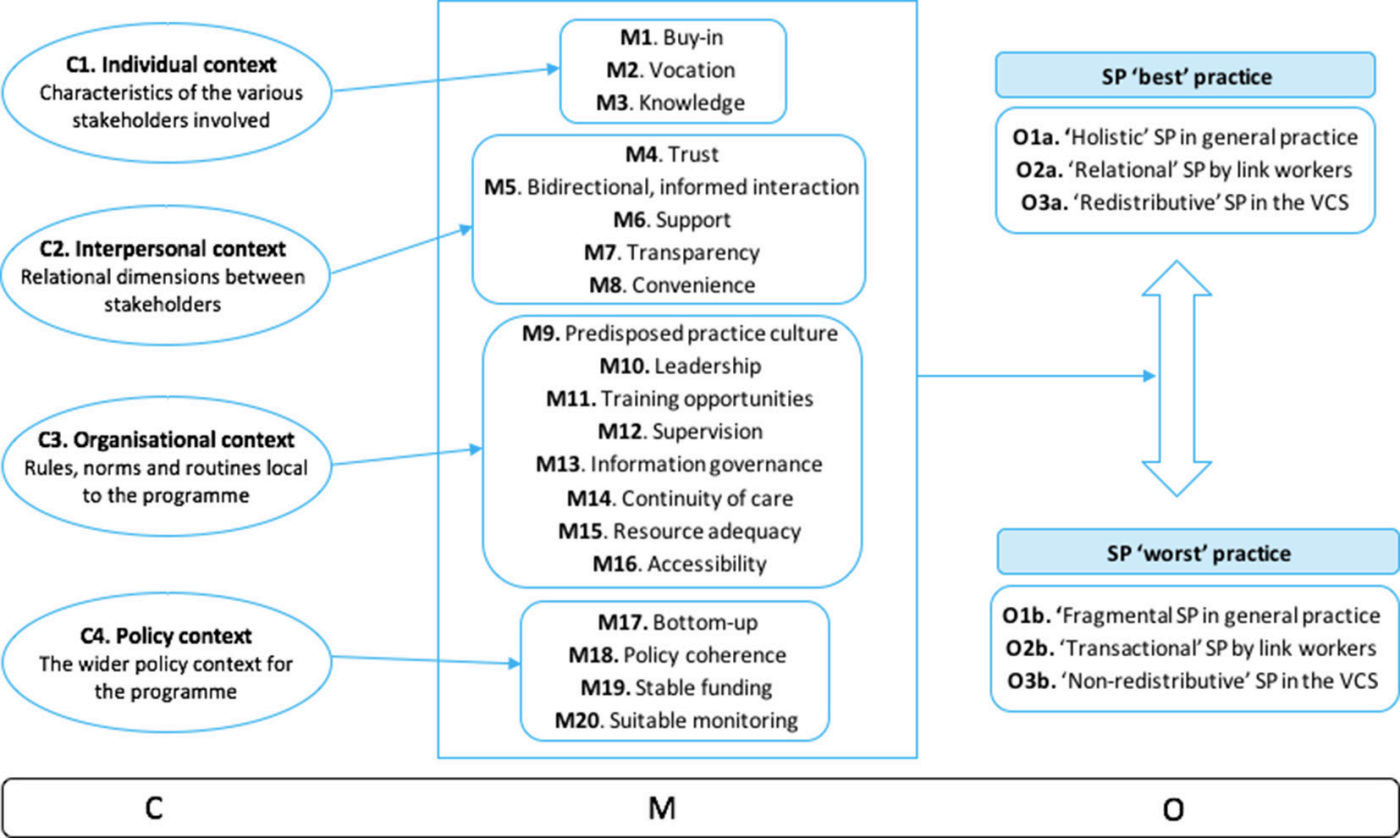
Context (C) mechanism (M) outcomes (O) identified in the literature reviewed. SP = social prescribing. VCS = voluntary and community sector.

Using a realist approach, it was explored how resources and conditions (mechanisms) available in specific individual, interpersonal, organisational, and policy environments (context) make these archetypical practices (outcomes) more (or less) likely. Such an analysis enabled the authors to build broad sets of CMOCs as represented in Figure 2 and described with data extracts in Supplementary Table S3 and extensively in Supplementary Appendix S4. In the following section, the identified contexts and mechanisms that facilitate ‘holistic’, ‘relational’, and ‘redistributive’ SP practices are expanded on.

### Individual characteristics

People are not ‘passive recipients of innovations’.^36^ Rather, they have values, preconceptions, allegiances, and commitments that influence their response to innovations. If general practice workers have significant ‘buy-in’ towards SP and believe that link workers and VCS organisations can play a role in addressing patients’ needs, it is more likely that they will engage in collaborative work and undertake a holistic SP.^37–39^ Likewise, link workers’ and community stakeholders’ previous life and work experience seems relevant in equipping them with valuable skills that facilitate SP delivery, such as knowledge of available initiatives, communication, and a person-centric approach.^40^ Their level of vocation also increases the dedication and commitment towards the service.^41^

General practice and link workers’ degree of knowledge of patients' circumstances, the SP scheme, and the local community organisations seem key in prompting informed discussions with patients and appropriate referrals.^39,42–48^ Link workers’ area of expertise may extend to welfare support services, which contributes to widen the scope of the intervention by potentially addressing socioeconomic concerns (instead of narrowly focusing the intervention on lifestyle issues).^2^ Through the understanding and acknowledgement of the specific contexts and challenges that different stakeholders face, link workers seem to be able to ‘negotiate the communication’ across sectors and bring them closer.^46^

### Interpersonal relations

Trust is developed through sustained, unhurried, and non-judgemental relationships, and seems to be a prominent mechanism for patient engagement, satisfaction, and partnership sustainability.^44,48^ Personalised interactions allow the design of interventions tailored to patients’ needs, enhancing service appropriateness.^18,49^ Although informed discussions with patients (concerning the referral process and the characteristics of the activities on offer) help to accommodate expectations^18,50^ and temper the ‘fear of the unknown’,^19^ additional emotional and practical support is often needed to overcome (or cope with) the barriers that prevent uptake and engagement.^47,48,50–54^ Patients are more likely to participate when link workers contact them directly after receiving the referral, make regular follow-up phone calls, or even come along with them to the planned activities.^49,55^ In the community, ongoing supervision by activity leaders is identified as a relevant factor promoting service users’ adherence.^56–60^ Support from peers in similar circumstances also enhances patients’ motivation by providing positive exemplars of progress and contributes to validate their personal experiences.^61–63^

Across sectors, collaboration can often be threatened by lack of trust, especially where link workers and the VCS are not considered as an appropriate route to addressing patients’ needs, or when one party interacts with the other for an ulterior and/or covert motive (also referred to as ‘strategic action’).^64–66^ Regular feedback to referring clinicians provides reassurance, encourages further referrals, and improves the way in which the service is used.^67,68^ Additionally*,* if the initial contact with the SP programme is easy and simple for primary care workers (for example, IT integration, lack of red tape, single point of contact, and/or physical co-location), it is more likely that they will initiate collaborative work and share relevant information on patients’ needs and background with link workers and community organisations.^53,66^ Practice staff should, however, be able to develop and refine their own referral system so that it can fit into existing practice routines and preferences. The system becomes convenient to the extent that it adapts to new challenges and is customised to workplace specificities.^69^

### Organisational contingencies

The shared beliefs, priorities, and values of the primary care team as a whole (practice culture) influence the attitude of individual members.^68^ Some primary care workers may be more strategically placed than others and, hence, have greater capacity to drive SP forward. The endorsement of the programme by GPs, for instance, gives credibility to the scheme and increases other professionals’ engagement because of their *‘professional and social standing’*.^70^

Training opportunities within primary care organisations also increase workers ‘capability’ to successfully incorporate SP into daily practice.^66,71^ In order for learning to be purposeful and applicable to day-to-day work, it should combine the concrete and practical experience with discussion with peers.^8^ An environment offering supervision and peer support allows link workers, for instance, to discuss difﬁcult patients and learn from challenging situations.^40,41^ Training of the VCS staff equates to service quality for primary care stakeholders and increases their reliability on the service.^19,65^

Across sectors, integrated information governance strategies and ongoing access to regular care providers seem key in enabling connected and coherent SP services.^38,70^ The availability of a named link worker connected to a geographical area (including the GP practices, patients, and community organisations within its remit) facilitates the embedding of the service within the local primary care infrastructure,^72^ and the development of knowledge on (and engagement with) support services within the local community.^70^ SP users value knowing that support is available ‘*for when it* [is] *needed’*.^55^ This ‘open door’ nature of the SP service allow link workers to provide ongoing care, experience patients’ changing circumstances and needs, and adapt relevant services accordingly.^69^

Conversely, increased workload and time pressures in primary care lead health professionals to prioritise patients’ specific reasons for consultation and/or incentivised activities, making it difficult to bring alternative community-based approaches into the conversation.^47,73^ Insufficient link worker staffing levels can lead to long waiting times to be assessed and subsequently pose a greater risk for non-engagement.^74^ Within a context of scarce resources, link workers may end up in prioritising immediate and urgent demands (*‘* *”* *fire-fighting* *“* *approach*’^46^), and not have enough capacity for innovative community engagement initiatives or to support individuals with enduring and complex health and social needs.^18^ As for the VCS, resource availability should be aligned with service demand, otherwise SP activities may end up congested and/or less accessible.^47,75^ Access to the VCS is also determined by the cost,^76^ timing,^62^ location,^44^ variety,^7^ and social and cultural appropriateness^77^ of the activities, and is both a condition for equity and a service-quality component.^78^

### Policy context

Bottom-up policymaking approaches tend to emphasise participation, making it easier for local communities to raise their concerns, prioritise goals, and select the means of achieving them.^79^ Supporting policy and guidance that are receptive to local knowledge, and boost existing capacities and autonomy tend to enhance ownership and the ‘embedded’ nature of change.^68,72^ Likewise, mutually reinforcing policy actions undertaken across different departments and agencies are more likely to create synergies towards ‘holistic’, ‘relational’, and ‘redistributive’ SP. This involves developing policy strategies targeted at strengthening each of the three settings (general practice, link workers, and the VCS). Representatives from the VCS, however, often raise concerns over the ‘*unprecedented’* level of budget deficits for social care and community organisations, which affect the sustainability and capacity of their services.^3,47^ Similarly, general practice workers often highlight underinvestment in primary care and resulting increased workload as main barriers to appropriate service delivery.^69,70^ The often short-term nature of contracts and the constant threat of funding withdrawal or reduction lead to significant turnover of the workforce^66^ and the activities being delivered.^3^ This has a consequential negative impact on stakeholders’ expectations and commitment towards the service.^57,69^

Payments to SP providers, as well as service evaluation, often rely on monitoring metrics that should prove relevant (so they can be used to improve local services and not just to enforce the contract), flexible (so they can be negotiated and adapted to local circumstances and emerging approaches), and feasible (so they can be attained and provided without excessive administrative burden). However, Lowe *et al* provide evidence on how the process of generating data to meet the performance indicators can distort practice, be resource intensive, stressful, and encourage ‘creaming’ of clients (working with certain patient groups considered easiest to help).^80^ Further studies highlight that excessive administrative burdens may create challenges, particularly for smaller organisations and those with limited capacity.^81,82^ Where payments are tightly linked to the achievement of predefined targets (for example, targets related to health service utilisations, numbers of people recruited onto the programme, and the speed with which referrals are seen) and provider success defined by such metrics, it is more difficult to develop trustful relationships across sectors^82^ and use data for reflective learning and discussion.^83^

### Synthesis: developing a multilevel, dynamic, and usable SP Framework

**Figure 3.**
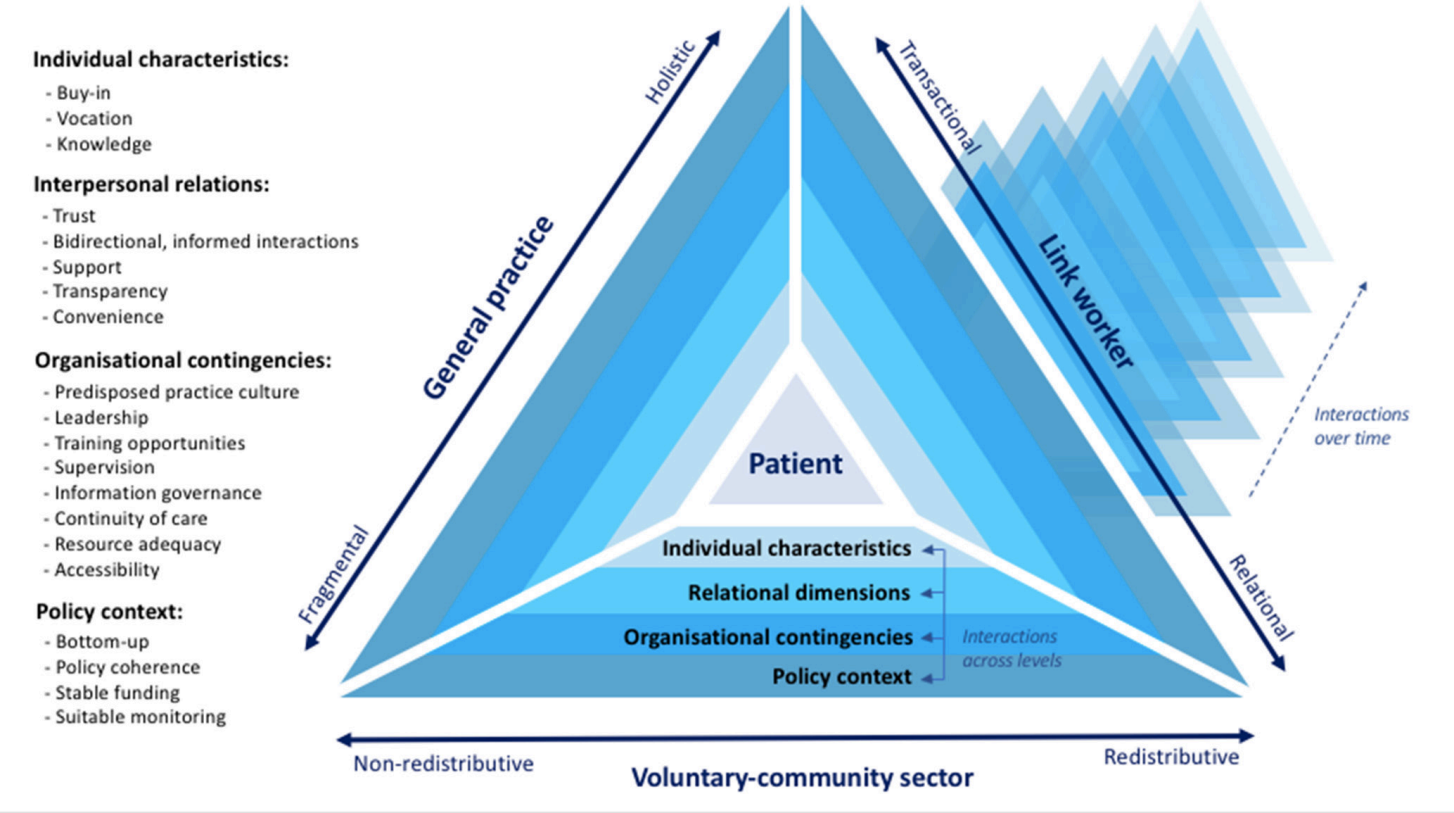
A Framework for Theorising and Evaluating Social Prescribing in Primary Care

The proposed Framework for Theorising and Evaluating SP in Primary Care is shown in Figure 3. It comprises three settings (general practice, link workers, and the VCS) and the previously described (see Figure 2) 20 mechanisms (buy-in, vocation, knowledge, trust, bidirectional and informed interactions, support, transparency, convenience, predisposed practice culture, leadership, training opportunities, supervision, information governance, continuity of care, resource adequacy, accessibility, bottom-up policymaking, policy coherence, stable funding, and suitable monitoring) in four interconnected contexts (individual, interpersonal, organisational, and policy).

The four interlinked arrows highlight the dynamic nature of the framework by illustrating how different mechanisms may relate to each other and mutually reinforce one another within and across contexts. The existence of a ‘social ethos of SP’ within the practice (referred to as ‘predisposed practice culture’ at the organisational level), for instance, seems to foster acceptance and enthusiasm among general practice workers (‘buy in’ at the individual level).^68^ Similarly, general practice workers’ understanding of the scheme (‘knowledge’ at the individual level) is enhanced by the availability of accessible and updated directories of VCS resources (‘information governance’ at the organisational level) and regular feedbacks from link workers (‘bidirectional and informed interactions' at the interpersonal level).^65^

The overlapping of layers represents the adaptation of and interaction between the different mechanisms over time. Primary care workers’ ‘knowledge’, for instance, is nurtured through the accumulation of episodic encounters that provide clinicians and link workers with relevant personal information about the patient and their context.^44,71,84^ The range of activities available through SP (‘accessibility’ at the organisational level) may also widen over time, as link workers iteratively adapt existing resources to patients’ needs and develop new activities where deficiencies are identified.^85^ The progression of the framework over time cannot, however, be predicted in advance and remains necessarily open. Indistinct shadows have been used to represent its non-linear nature and potential unintended consequences.

## Discussion

### Summary

Building on the reviewed literature, this study has developed a new framework. It allows for the characterisation of different (and often conflicting) SP practices, and the identification of conditions and resources across settings (general practice, link workers, and VCS) and systems (micro, meso, and macro), which contribute to ‘good’ SP practice in primary care.

### Strengths and limitations

The framework has been developed systematically, following rigorous methodological guidance for realist reviews as described in the RAMESES quality standards.^21,22^ The iterative nature of the realist approach has enhanced the scope and practical relevance of the framework, by incorporating relevant studies that would have not been identified through predefined search strategies. In addition, the consideration of different and interrelated settings and systems has allowed the analysis of SP practices in all their complexity and divergence, potentially increasing the applicability and transferability of findings. Limitations include being reliant on the evidence that is available. Some studies narrowly focused on intervention effectiveness (and magnitude of effect), without providing enough detail on how these results had been achieved, and, therefore, could not contribute to programme theory development or refinement.

### Comparison with existing literature

Previous studies have explored the role of SP in enhancing patients’ wellbeing and collaboration across sectors, and identified relevant preconditions to intended outcomes.^3,7,18,50,66,70^ The review extends beyond lists of barriers and facilitators to critically understand what ‘good’ practice in SP looks like, and how and in what contexts this might be best achieved. The paired archetypes reveal that SP is not inherently advantageous. In the absence of specific individual, interpersonal, organisational, and policy resources, interventions could adversely lead to the fragmentation of primary care services, stakeholder disengagement, and greater health inequalities.

Relevant individual characteristics have been identified, such as the degree of ‘buy in’, vocation, and knowledge, which influence not only stakeholders’ attitude towards SP, but also the way in which they use, consolidate, or even modify the intervention. The review and resulting framework also highlight that SP does not happen in a vacuum, but it is rather developed, sustained, and shaped by a dynamic set of interactions across and within sectors. Mutual reliance and the development of trustful, supportive, and ongoing relationships seem central to the success of SP interventions.^2,44,47,65,69,86^ This idea of interdependence conflicts with the prevalent representation of SP programmes as unidirectional and linear referral pathways towards patient’s ‘activation’ or ‘independence’.^3,87^ The triangle-shaped framework conceptualises SP as a network comprising multiple kinds of relationships (therapeutic, administrative, and professional) that potentially link stakeholders (general practice workers, link workers, and members of the VCS) with one another in an overarching, integrated, and ongoing purpose of caring for the patient being referred. The findings could help to explain why evaluative approaches that conceptualise and measure effectiveness in terms of reduced service utilisation (as a proxy for patients’ activation^88^) often fail to prove the value and potential impact of SP.

The identified meso and macro level dimensions highlight that neither SP nor the individuals who deliver and use the service can be studied effectively in isolation from the complex organisational, social, and policy contexts in which they are embedded. Previous studies on SP have consistently recognised relevant organisational resources for effective service implementation, such as the availability of a vibrant VCS, information governance arrangements, or a conducive institutional ethos.^47,68,70,89^ In this case, the input from the stakeholder group and the additional literature search enabled these dimensions to be expanded on by also characterising and incorporating higher-level policy contexts, priorities, and decisions (‘bottom-up policymaking’, ‘stable funding’, ‘suitable monitoring’, and ‘policy coherence’) that shape SP delivery.

### Implications for research and practice

This study generates an actionable framework for SP implementation and evaluation, readily available for end users and policymakers. It builds on a burgeoning body of evidence on complex-system approaches to evaluation, by identifying outcomes and potential actions across sectors and at different levels.^14,15,90^ It is anticipated that this evidence-based, theory-informed framework could prove particularly useful for the design and roll-out of new SP interventions, and to identify relevant features (at micro, meso, and macro levels) that could facilitate optimisation of existent programmes. The framework is not intended to be used as a checklist when implementing or evaluating SP programmes. Rather, it is believed it could guide, inform, and potentially predict (but never in a deterministic way) ‘localised’ and ‘contextually sensitive’ implementation and evaluative efforts.

The multilevel framework arising from this realist review can also be usefully tested, refined, and expanded by applying it to specific patient groups. The researchers involved in this study are already exploring the applicability of the framework in populations at high risk of type 2 diabetes, and where existing preventive interventions have low uptake.^20^ The authors strongly encourage other research groups to explore the applicability of the framework in different settings and areas of health need, and to adapt and extend it as appropriate.

## Trial registration number

PROSPERO: CRD42020196259.
